# Successful use of the sodium-glucose co-transporter-2 inhibitor dapagliflozin in patients with renal transplant and diabetes: a case series and literature review

**DOI:** 10.1097/XCE.0000000000000246

**Published:** 2021-03-17

**Authors:** Wajiha Gul, Emad Naem, Safa Elawad, Tarik Elhadd

**Affiliations:** a Department of Medicine, Endocrinology Section & Qatar Metabolic Institute, Hamad Medical Corporation; b Department of Medicine, Section of Nephrology, Hamad General Hospital, Doha, Qatar

**Keywords:** diabetes, glycated hemoglobin, hyperglycemia, renal transplant, sodium-glucose co-transporter-2 inhibitor

## Abstract

Management of patients with diabetes and renal transplant could be challenging. Transplant patients use multiple immune suppressants that can worsen or even trigger hyperglycemia. There are no data about the use of the new class of sodium-glucose co-transporter-2 (SGLT-2) inhibitor dapagliflozin in patients with renal transplant and diabetes.

**Case series:**

Four patients, with diabetes, who are attending the diabetes clinic at our institution, are presented here. They were all counseled to be started on dapagliflozin 10 mg to improve diabetes control as they were on multiple agents and not achieving targets. All four patients showed significant improvement in hemoglobin A1c, with no adverse effects on renal parameters and had favorable effect on weight and blood pressure (BP).

**Conclusion:**

Use of the SGLT-2 inhibitor dapagliflozin in the standard dose of 10 mg helped to achieve satisfactory control with favorable effects on BP and weight with no adverse effects on renal function.

## Introduction

Type 2 diabetes is most prevalent in patients undergoing renal transplant. Post-transplant diabetes mellitus (PTDM) is a separate entity that develops after solid organ transplantation in patients with no prior history of diabetes [1]. It is also known as new-onset diabetes after transplant (NODAT) and is associated with significant mortality and morbidity. Type 2 diabetes mellitus and PTDM are two different pathophysiological processes; however, so far they are treated on the same lines of management due to a lack of significant data and studies on hyperglycemia developing post-solid organ transplantation [2]. PTDM is different from type 2 diabetes in various aspects. Post-transplant care includes the use of immunosuppressant medications and steroids that are potentially diabetogenic affecting both insulin sensitivity and insulin secretion. Risk of viral infections as cytomegalovirus, hepatitis C, and so on also plays a significant role [3–6].

Sodium-glucose co-transporter-2 (SGLT-2) inhibitors are emerging as preferred treatment options for patients with cardiovascular comorbidities and renal impairment because of their cardioprotective and nephroprotective effects as established by recent studies. However, their use in PTDM is still under discussion. There is no consensus for their use as first-line medication in renal transplant patients. There are few reports in which SGLT-2 inhibitor empagliflozin use resulted in statistically significant reduction in hemoglobin A1c (HbA1c), weight loss, stable allograft function, and improvement in SBP with no notable adverse effects. Shah *et al*. [7] published about the safety and efficacy of canagliflozin in 24 patients with renal transplant and observed improved glycemic control with an increase in the rate of infections. Another study by Kong *et al*. [8] published the efficacy of dapagliflozin in 42 kidney transplant recipients with no major adverse effects, of these 42 patients, 14 had PTDM.

We present here a series of renal transplant cases with both pre-existing diabetes and PTDM that were treated with dapagliflozin at our tertiary care institute with good clinical outcomes.

## Case series

### Case 1

A 54-year-old man originally from the Indian Subcontinent was diagnosed with type 2 diabetes mellitus in 2001 complicated by end-stage renal disease underwent live unrelated renal transplant in 2013. He was on high doses of premixed insulin and a combo preparation of sitagliptin and metformin (Janumet). Diabetes remained suboptimally controlled despite close follow-up and insulin titration in the post-transplant period. HbA1c remained in the range of 8.5–9%. He had other complications of diabetes like diabetic retinopathy and painful diabetic neuropathy. He also had hypertension and hyperlipidemia and was appropriately treated for his comorbid conditions. The allograft function was stable with no proteinuria. In 2018, we elected to give him trial of SGLT-2 inhibitor dapagliflozin 10 mg daily after explaining in detail the pros and cons of the new medicine. He was advised to continue on his previous regimen (premixed insulin and Janumet) along with his other antidiabetic medications. HbA1c improved from 8.5% in November 2017 to 6.2% in March 2018. Six months later, upon his follow-up in his home country, his transplant surgeon stopped dapagliflozin as he was not comfortable with the medication. This resulted in rise in HbA1c to 9.2%. Later dapagliflozin was resumed and his glycated hemoglobin remained in the range of 6.5–7.0%. He had other benefits in terms of weight loss as his weight improved from 95 to 92 kg and then remained stable between 92 and 93 kg even a year later. To date, there were no reported adverse effects.

### Case 2

A 49-year-old Qatari woman developed PTDM in 2008 following renal transplant which was done in 2007. She was initially on basal insulin with oral medications [insulin glargine, gliclazide 60 mg daily, and Janumet XR (sitagliptin/metformin) 50/1000 mg two tablets daily]. In July 2017, her glycated hemoglobin was 7.5% so it was decided to be started on dapagliflozin 10 mg daily. Other comorbidities include hypertension and dyslipidemia. She was followed up closely and at follow-up visit 6 months later her HbA1c was 6.6% with no episodes of hypoglycemia. Her insulin requirements decreased. She remained on the above treatment regimen. She remained well on her new treatment. There were favorable effect on her weight and BP, with weight improved from 56 to 50.5 kg and remained between 50 and 52 kg. She had initially one episode of urinary tract infection that was treated successfully with oral antibiotics and she remained well thereafter.

### Case 3

A 58-year-old Sudanese man with a history of renal transplant in 2012 (live, unrelated) developed PTDM/NODAT in 2013. He was treated with basal insulin and oral medications [gliclazide 120 mg once daily and Janumet (sitagliptin/metformin XR) 50 mg/1000 mg twice daily]. He remained suboptimally controlled; however, the renal allograft was functioning well and there was no episode of rejection. In July 2017, the SGLT-2 inhibitor dapagliflozin 10 mg was added. His blood glucose improved significantly and HbA1c improved from 8.1 to 7% after 6 months. He has to discontinue insulin and gliclazide. Currently, he is doing well on Janumet and dapagliflozin. There was no episode of graft rejection and no adverse effects happened.

### Case 4

A 66 years old male of Iranian descent with renal transplant in 2014 secondary to renal calculi and obstructive uropathy. His other comorbidities include hypertension and coronary artery disease. He developed diabetes in 2018 and was initially started on insulin glargine, insulin aspart, and metformin. His HbA1c in September 2018 was 11%. He was then started on oral medications which include vildagliptin and dapagliflozin along with basal insulin glargine. His glycemic control improved significantly with HbA1c dropping to 6.4% from 11%.

**Table:** Summary of the cases

	Characteristics	Case 1	Case 2	Case 3	Case 4
1	Age in years	54	49	58	66
2	Gender (male/female)	Male	Female	Male	Male
3	Kidney donor (live/cadaveric)	Live, unrelated		Live, unrelated	
4	Time since renal transplant	7 years	13 years	7 years	5 years
5	Type of diabetes	Type 2 DM	PTDM	PTDM	PTDM
6	Time since diabetes	19 years	12 years	6 years	2 years
7	Baseline body weight (kg)/BMI	95 (28.68)	56 (24.8)	95.30 (29.64)	140 (48.4)
8	Weight after 1 year (kg)/BMI	93 (28.08)	50.5 (22.7)	91 (28.4)	122 (41.7)
9	Adverse effects:				
	(i) Allograft dysfunction	Nil	Nil	Nil	Nil
	(ii) DKA	Nil	Nil	Nil	Nil
	(iii) Infections (genital/urinary)	Nil	I episode, improved with antibiotics	Nil	Nil
10	Any event leading to drug discontinuation	No	No	No	No

DKA, diabetic ketoacidosis.

## Discussion

NODAT or PTDM is associated with acute transplant rejection, infections, late cardiovascular events, and decreased overall survival. This raises the need for optimal glycemic control. SGLT-2 are located at the proximal tubules of kidney and act to absorb glucose from glomerular filtrate [9]. Substantial inhibition of these transporters leads to the secretion of excess glucose into the urine and this effect is independent of insulin levels in the blood [9]. The renoprotective and cardioprotective effects of SGLT-2 inhibitors have been established by large clinical trials in type 2 diabetes patients [10–12]; however, the beneficial effects of these medications still need to be studied in solid organ transplant recipients.

A study published by Halden *et al*. [13] in March 2019 in Diabetes Care looked at the efficacy and safety of empagliflozin in renal transplant patients with PTDM. It was a prospective, single-center, double-blind study in which two groups of patients (total of 49 participants of which 44 completed the study) were randomized to receive either placebo or empagliflozin 10 mg. There was a significant improvement in glycemic control and body weight with no notable difference in adverse events, estimated glomerular filtration rate, or immunosuppressant drug levels [13]. Empagliflozin was also studied in cardiac transplant patients with PTDM by Cehic *et al*. [14] in which they studied 22 patients. Empagliflozin was well tolerated by all patients with no adverse effect of genitourinary infections [14].

A pilot study published by Shah *et al*. [7] assessed the effects of canagliflozin in 24 renal transplant patients with either pre-existing type 2 diabetes or PTDM. They observed improvement in glycemic control with the need to reduce the dose of insulin/oral hypoglycemic agents along with improvement in body weight (mean weight loss of 2.4 kg in 12 weeks), BP (both systolic and diastolic around 8/2 mmHg) with stable renal function and tacrolimus trough levels [7]. The adverse effects such as increased urination, urinary tract infection, and genital mycotic infections were not observed in any of the patients [7].

Drug–drug interaction was assessed in patients taking dapagliflozin and immunosuppressant medications. Wallia *et al*. [15] observed no pharmacokinetic interaction between canagliflozin and cyclosporine likely because of different mechanisms of action. Metabolism of the SGLT 2 inhibitor, dapagliflozin takes place mainly in the liver and kidneys by uridine diphosphate-glucuronyltransferase-1A to its metabolite dapagliflozin 3-*O*-glucuronide [16] while empagliflozin mainly has hepatic glucuronidation [14]. mammalian target of rapamycin inhibitors and calcineurin inhibitors are metabolized by P450 enzymes [14].

We gave a trial of dapagliflozin to four patients with the renal transplant, three of whom had PTDM and one had type 2 diabetes mellitus. All these patients were started on dapagliflozin after detailed discussion and counseling with the pros and cons were fully covered. All these patients responded significantly to dapagliflozin with no significant adverse events that could have led to medication discontinuation. None of the patients had an episode of genital infections. Only one patient had a urinary tract infection that was treated appropriately and she responded well to it. However, she preferred to continue her medication and did not have any episodes afterwards. No episode of any diabetic ketoacidosis. All of our patients had stable allograft function. There was no case of deterioration in renal parameters following the initiation of dapagliflozin. All patients had optimal levels of immunosuppressant medications with no interference observed in the pharmacokinetics of these medications and no episode of graft rejection was seen. These patients were in close follow-up with the nephrology team. There was significantly favorable effect on weight which was around 2–3% of their body weight.

### Conclusion

To the best of our knowledge, this is the first case series of patients with renal transplant and diabetes mellitus (both type 2 diabetes mellitus and PTDM/NODAT) in which dapagliflozin was used with significant effects on glycemic control, bodyweight without any deterioration in allograft dysfunction or interference with the pharmacokinetics of immunosuppressant medications. More data are needed to establish the cardiovascular benefits and renoprotective effects of SGLT-2 inhibitors in these patients.

## Acknowledgements

This article is supported by Medical Research Council, Hamad Medical Corporation.

## Conflicts of interest

There are no conflicts of interest.

## References

[1] Jenssen T Hartmann A. Post-transplant diabetes mellitus in patients with solid organ transplants. Nat Rev Endocrinol 2019; 15:172–188. 30622369 10.1038/s41574-018-0137-7

[2] Hecking M Jenssen T. Considerations for SGLT2 inhibitor use in post-transplantation diabetes. Nat Rev Nephrol 2019; 15:525–526. 31235880 10.1038/s41581-019-0173-0

[3] Heit JJ. Calcineurin/NFAT signaling in the beta-cell: From diabetes to new therapeutics. Bioessays 2007; 29:1011–1021. 17876792 10.1002/bies.20644

[4] Hjelmesæth J Müller F Jenssen T Rollag H Sagedal S Hartmann A. Is there a link between cytomegalovirus infection and new-onset posttransplantation diabetes mellitus? Potential mechanisms of virus induced β-cell damage. Nephrology Dialysis Transplantation 2005; 20:2311–2315. 10.1093/ndt/gfi033 16046502

[5] Hecking M Kainz A Werzowa J Haidinger M Döller D Tura A. Glucose metabolism after renal transplantation. Diabetes Care 2013; 36:2763–2771. 23656979 10.2337/dc12-2441 PMC3747896

[6] Jørgensen MB Hornum M van Hall G Bistrup C Hansen JM Mathiesen ER Feldt-Rasmussen B. The impact of kidney transplantation on insulin sensitivity. Transpl Int 2017; 30:295–304. 28000288 10.1111/tri.12907

[7] Shah M. Efficacy and safety of canagliflozin in kidney transplant patients with type 2 diabetes. Indian J Transplantation 2016; 4:85. 10.4103/ijn.IJN_2_18 PMC6668319 31423063

[8] Kong J Joon J Chul Y Eun W Hyuk K Sung Hyun S. SP770 sodium/glucose cotransporter 2 inhibitor for the treatment of diabetes in kidney transplant patients. Nephrology Dialysis Transplantation 2019; 34(Supplement_1):gfz103–SP770.

[9] Gallo LA Wright EM Vallon V. Probing SGLT2 as a therapeutic target for diabetes: basic physiology and consequences. Diab Vasc Dis Res 2015; 12:78–89. 25616707 10.1177/1479164114561992 PMC5886707

[10] Neal B Perkovic V Mahaffey KW de Zeeuw D Fulcher G Erondu N. Canagliflozin and cardiovascular and renal events in type 2 diabetes. N Engl J Med 2017; 377:644–657. 28605608 10.1056/NEJMoa1611925

[11] Levin A Perkovic V Wheeler DC Hantel S George JT von Eynatten M. Empagliflozin and cardiovascular and kidney outcomes across KDIGO risk categories: post hoc analysis of a randomized, double-blind, placebo-controlled, multinational trial. Clin J Am Soc Nephrol 2020; 15:1433–1444. 32994159 10.2215/CJN.14901219 PMC7536760

[12] Heerspink HJL Stefánsson BV Correa-Rotter R Chertow GM Greene T Hou F. Dapagliflozin in patients with chronic kidney disease. N Engl J Med 2020; 383:1436–1446. 32970396 10.1056/NEJMoa2024816

[13] Halden TAS Kvitne KE Midtvedt K Rajakumar L Robertsen I Brox J. Efficacy and safety of empagliflozin in renal transplant recipients with posttransplant diabetes mellitus. Diabetes Care 2019; 42:1067–1074. 30862658 10.2337/dc19-0093

[14] Cehic MG Muir CA Greenfield JR Hayward C Jabbour A Keogh A. Efficacy and safety of empagliflozin in the management of diabetes mellitus in heart transplant recipients. Transplant Direct 2019; 5:e450. 31165085 10.1097/TXD.0000000000000885 PMC6511439

[15] Wallia A Illuri V Molitch ME. Diabetes care after transplant: definitions, risk factors, and clinical management. Med Clin North Am 2016; 100:535–550. 27095644 10.1016/j.mcna.2016.01.005

[16] Kasichayanula S Liu X Lacreta F Griffen SC Boulton DW. Clinical pharmacokinetics and pharmacodynamics of dapagliflozin, a selective inhibitor of sodium-glucose co-transporter type 2. Clin Pharmacokinet 2014; 53:17–27. 24105299 10.1007/s40262-013-0104-3

